# An Atypical Pediatric Dermatologic Presentation Unmasking an Unexpected Systemic Diagnosis: A Diagnostic Challenge

**DOI:** 10.7759/cureus.103019

**Published:** 2026-02-05

**Authors:** Brooke Heyer, Leonardo Bonifanti, David R Jetha, Cristina Figallo

**Affiliations:** 1 Department of Medicine, Dr. Kiran C. Patel College of Osteopathic Medicine, Nova Southeastern University, Fort Lauderdale, USA; 2 Department of Pediatrics, Broward Health Medical Center, Fort Lauderdale, USA

**Keywords:** dermatology, epidemiology, hiv, iga vasculitis, pediatrics

## Abstract

Dermatologic and musculoskeletal presentations in pediatric patients can pose significant diagnostic challenges, particularly when findings are atypical, persistent, and resistant to therapy. We present a diagnostically complex case of a 13-year-old male with a history of multiple psychiatric comorbidities who developed progressive bilateral pedal edema, bullous lower extremity lesions with excoriations, and severe pain, all symptoms seemingly refractory to antibiotics. Despite initial concern for skin and soft-tissue infection, symptoms persisted, prompting inpatient evaluation and an expanded serologic workup. Screening for immunologic and infectious causes, including human immunodeficiency virus (HIV) and sexually transmitted infections, revealed a new diagnosis of HIV-1 acquired through non-perinatal transmission. The patient was concurrently diagnosed with atypical IgA vasculitis, raising consideration of infectious versus drug-related triggers based on his medication regimen. Although vasculitis is uncommon in HIV-positive patients, and IgA vasculitis is even rarer, this case emphasizes the importance of a broad differential diagnosis in pediatric patients with multisystem involvement. Ultimately, close attention to atypical presentations can ensure timely diagnoses and better care for patients facing similarly complex conditions.

## Introduction

Cutaneous manifestations in pediatric and adolescent populations can pose significant diagnostic challenges, particularly when lesions are persistent, progressive, atypically distributed, and refractory to standard therapies. While bacterial, autoimmune, and hypersensitivity etiologies often top the differential diagnosis, broader systemic or less common underlying conditions may be overlooked, especially in younger populations or in patients who appear otherwise healthy.

Human immunodeficiency virus (HIV) is a global disease that causes progressive CD4^+^ T-cell depletion and increases susceptibility to malignancy, opportunistic infections, and multisystem dysfunction due to immune suppression [1]. Systemic vasculitides, including IgA vasculitis (Henoch-Schönlein purpura {HSP}), represent rare but important manifestations of immune dysregulation and may present atypically in pediatric patients, particularly when underlying immunologic conditions are present. First-line management of HIV includes antiretroviral therapy as well as prophylaxis against opportunistic infections based on CD4^+^ count, both seen in our case. HIV transmission most commonly occurs either perinatally (vertical transmission) or in vulnerable adult populations, including intravenous drug users, men who have sex with men, and sex workers. Newly acquired HIV in pediatric or adolescent populations is significantly less common than perinatal or adult acquisition [1,2]. In the United States, approximately 25.7% of new HIV cases occur in individuals aged 13-24 years, and most are concentrated in the younger adult subgroup. Perinatal transmission still accounts for the majority of HIV cases in younger children, yet behavioral acquisition among adolescents and young adults continues to be a significant and growing public health concern [3]. When adolescents newly acquire HIV, their risk factors often differ from those of adults and may include childhood sexual abuse, trafficking, coercion, or age-related experimentation. While these factors were considered in this case as part of the acquisition assessment, none were explicitly confirmed, and their inclusion highlights broader patterns that contribute to the growing concern of behavioral HIV acquisition in youth. These factors may also complicate care by introducing legal and ethical complexities regarding reporting and the involvement of external agencies [4].

Certain populations are disproportionately affected, including Black/African American and Hispanic/Latino youth [3]. Despite this, testing rates remain low. One study found that only 12.9% of high school students and 34.5% of individuals aged 18-24 years had been tested for HIV, and testing rates were significantly lower among males than females, and lower among White and Hispanic/Latino populations than among Black/African American adolescents [3]. Because HIV is infrequent in this age group, it is often excluded from common diagnostic testing, which may delay identification in cases of patients with inconclusive or atypical examination findings. Physicians should be cautious not to dismiss diagnostic possibilities solely on the basis of statistical odds of disease in certain groups.

We present a diagnostically complex case of a 13-year-old male with progressive asymmetric pedal edema and ulcerative lower extremity bullous lesions that were unresponsive to empiric antimicrobial therapy. Diagnostic evaluation and management were complicated by behavioral and psychiatric comorbidities, contributing to delays in testing. Ultimately, expanded laboratory assessment recommendations, including sexually transmitted infection (STI) screening, revealed a new diagnosis of human immunodeficiency virus (HIV) acquired through non-perinatal transmission, reframing the diagnostic approach and guiding subsequent management.

This case highlights an unusual dermatologic and musculoskeletal presentation of newly diagnosed HIV in an adolescent, emphasizing the importance of maintaining a broad differential diagnosis when cutaneous and musculoskeletal findings mimic other etiologies. It emphasizes the need to consider HIV and STI screening in pediatric and adolescent patients with atypical or inconclusive presentations, particularly when standard therapies fail, and care is complicated by medical, psychiatric, or social factors.

## Case presentation

A 13-year-old Black male with a history of attention deficit hyperactivity disorder (ADHD), oppositional defiant disorder (ODD), bipolar disorder, and multiple psychiatric hospitalizations presented to his primary care physician (PCP) with bilateral foot pain, pedal swelling, and a rash that appeared one week prior on his lower extremities. He was prescribed cephalexin for presumed skin and soft-tissue infection, but his symptoms did not improve. He subsequently presented to the emergency department (ED) three days after his PCP visit with worsening lower extremity maculopapular lesions at varying stages of healing, difficulty ambulating, and tenderness on the soles of his feet. He had no fever but reported a three-day history of emesis and diarrhea. Examination revealed a non-pruritic, scaling, annular rash on both legs and the ankles, with associated swelling. Laboratory testing was unremarkable, aside from an elevated erythrocyte sedimentation rate (ESR) of 84 mm/h (normal: 0-10 mm/h), and X-rays of the lower extremities were within normal limits (Table 1). He was started on empirical treatment with clindamycin, mupirocin, and acetaminophen as needed, and was admitted for further workup.

**Table 1:** Laboratory findings on presentation. HSV: herpes simplex virus; CBC: complete blood count; CMP: comprehensive metabolic panel; AST: aspartate aminotransferase; ALT: alanine aminotransferase; CRP: C-reactive protein; ESR: erythrocyte sedimentation rate; ANA: antinuclear antibody; GIP: gastrointestinal pathogen panel; RPR: rapid plasma reagin; NAAT: nucleic acid amplification test

Laboratory tests	Patient values	Reference ranges
CBC		
White blood cell count (×10⁹/L)	4.06	4.5-11.0
Hemoglobin (g/dL)	12.7	13.5-17.5
Hematocrit (%)	40.70	38-50
Platelets (×10⁹/L)	290	150-400
Neutrophils (absolute) (×10⁹/L)	1.35	1.8-7.7
Lymphocytes (absolute) (×10⁹/L)	1.34	1.0-4.8
CMP		
Sodium (mmol/L)	139	135-145
Potassium (mmol/L)	3.8	3.5-5.0
Chloride (mmol/L)	106	98-107
Bicarbonate (CO₂) (mmol/L)	25	22-29
Blood urea nitrogen (mg/dL)	8	7-20
Creatinine (mg/dL)	0.7	0.5-1.0
Glucose (mg/dL)	82	70-99
Calcium (mg/dL)	9.6	8.5-10.5
AST (U/L)	20	10-40
ALT (U/L)	15	7-56
Alkaline phosphatase (U/L)	147	44-147
Total bilirubin (mg/dL)	0.4	0.2-1.2
Albumin (g/dL)	3.6	3.5-5.0
Inflammatory markers		
CRP (mg/dL)	0.81 (later down to 0.13)	<0.5
ESR (mm/h)	84 (later down to 16)	0-15
HIV-related studies		
HIV-1 antibody	Positive	Negative
HIV-1 RNA viral load (copies/mL)	669,000 (later down to 615)	Undetectable
HIV-1 RNA log_10_	5.83	Undetectable
CD4⁺ T-cell count (cells/µL)	216 (later up to 341)	500-1,500
CD8⁺ T-cell count (cells/µL)	371	150-1,000
CD4/CD8 ratio	0.58	1.0-4.0
Autoimmune/genetic testing		
ANA	Negative	Negative
HLA-B27 antigen	Positive	Negative
Infectious disease testing		
HSV-1/2 serologies	Negative	Negative
Gonorrhea (NAAT)	Negative	Negative
Chlamydia (NAAT)	Negative	Negative
Syphilis serology (RPR)	Negative	Negative
Hepatitis A	Negative	Negative
Hepatitis B	Negative	Negative
Hepatitis C	Negative	Negative
GIP		
Yersinia enterocolitica	Positive	Negative
Other bacterial, viral, and parasitic pathogens	Negative	Negative
Microbiology		
Wound culture	Rare skin flora isolated	No growth

Due to persistent symptoms and concern for a systemic or immune-mediated process, rheumatology was consulted during this admission. A broad serologic workup was recommended, including inflammatory markers, autoimmune panels, and infectious testing. As part of this workup, HIV and broad sexually transmitted infection (STI) screening were performed with family consent. HIV antigen/antibody (HIV Ag/Ab) testing returned positive, and confirmatory studies revealed HIV-1 with a viral load of 669,000 copies/mL (normal: <20 copies/mL) and a CD4^+^ T-cell count of 216 cells/µL (19.5%) (normal: 400-2100 cells/µL) (Table 1). A pediatric infectious disease physician was consulted and prescribed the patient bictegravir-emtricitabine-tenofovir alafenamide (Biktarvy) and trimethoprim-sulfamethoxazole (TMP-SMX or Bactrim) for *Pneumocystis jirovecii* pneumonia (PJP) prophylaxis, as his CD4^+^ T-cell count was close to acquired immunodeficiency syndrome (AIDS) defining (<200 cells/µL). Additional inflammatory laboratory tests revealed the patient is HLA-B27 positive and antinuclear antibody (ANA) negative, and his gastrointestinal PCR was positive for *Yersinia enterocolitica*, which was presumed to be the cause of his initial gastrointestinal symptoms. Although *Yersinia enterocolitica* is a known trigger for reactive arthritis, particularly in HLA-B27-positive patients, the absence of ongoing gastrointestinal symptoms and negative repeat gastrointestinal testing during subsequent admissions, along with later biopsy-confirmed findings, supported an alternative etiology. CT scan of the brain with contrast and renal ultrasound were normal, and MRI of the foot and further imaging studies were recommended but ultimately deferred due to the patient’s refusal to cooperate with procedures.

Approximately 10 days after admission, the rash began to spread to the patient's hands, with additional knuckle swelling. Dermatology was consulted, yet the patient refused a skin biopsy as he had procedural and needle anxiety and did not want to cooperate. Over time, the rash started to disappear and the swelling diminished; however, this broadened the differential diagnosis, including hypersensitivity reaction to Bactrim, reactive arthritis secondary to *Yersinia enterocolitica* infection, and malignancy such as Kaposi sarcoma, among others, leaving the diagnosis inconclusive.

Roughly five days after discharge, the patient returned to the ED with recurrent bilateral lower extremity swelling, pain, and worsening bullous lesions. He had left the ED against medical advice the night prior but returned the next day following an outpatient visit with his pediatric infectious disease specialist, who recommended direct admission for intravenous antibiotics and further testing. On this visit, he was afebrile and had no gastrointestinal or upper respiratory symptoms. He was started on intravenous acyclovir for herpes simplex virus (HSV) empiric therapy, which later came back negative, and intravenous ceftriaxone and clindamycin, in addition to his HIV regimen of Biktarvy and Bactrim.

**Figure 1:**
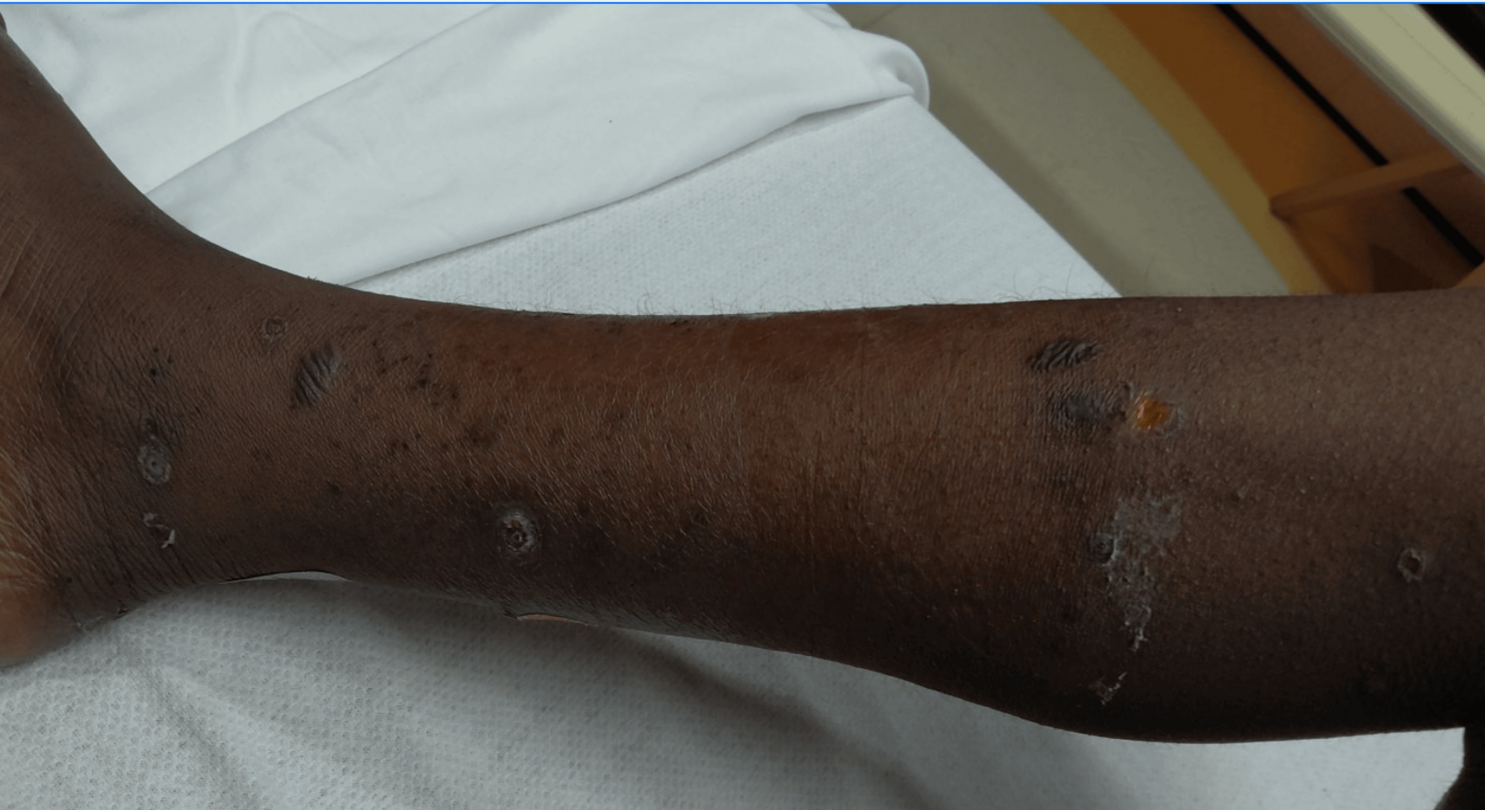
Right leg with few bullae and multiple ulcerated and encrusted lesions on lower leg and ankle at varying stages of healing.

**Figure 2:**
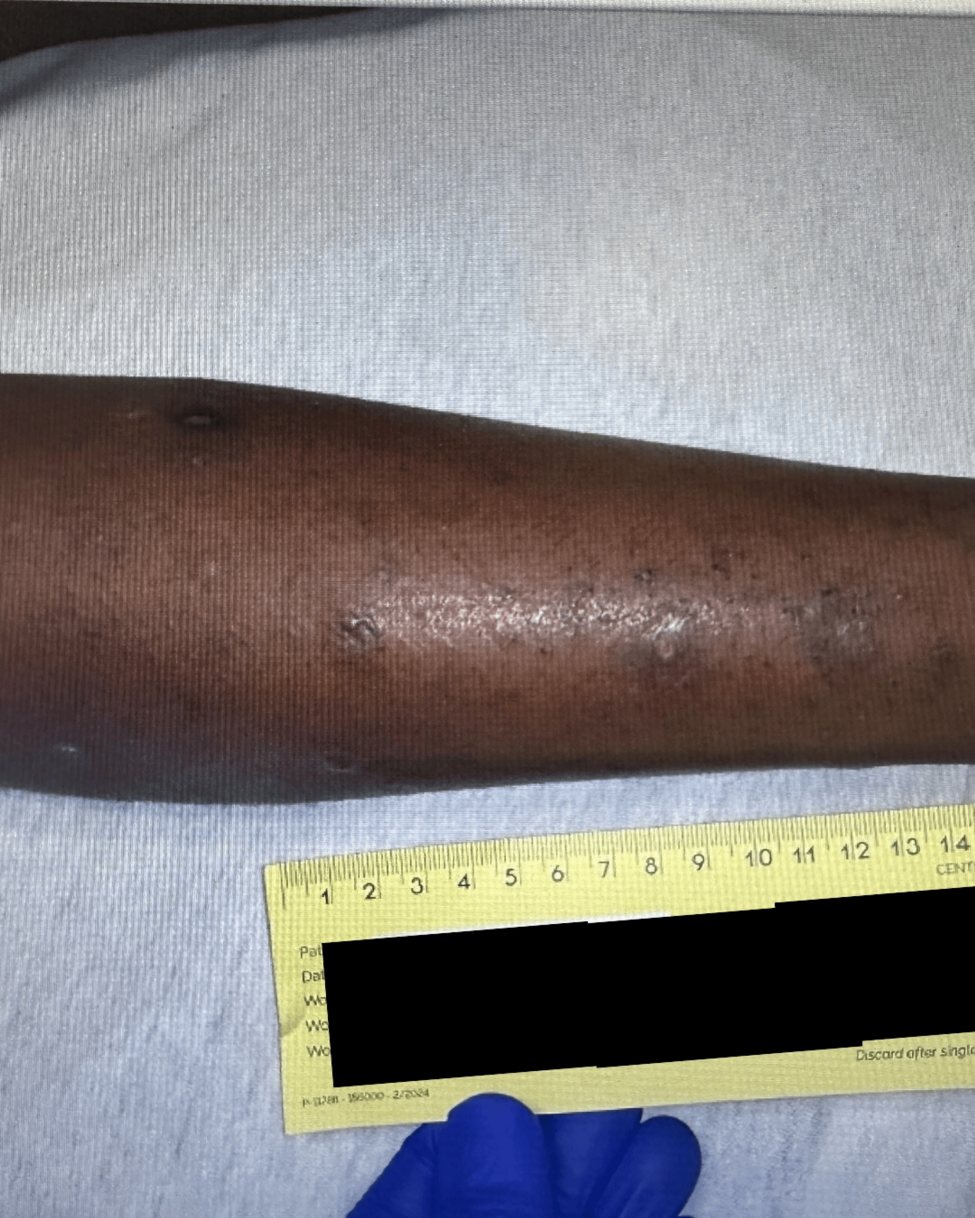
Another view of the lesions on the patient's lower extremity.

On the physical examination, the patient had bilateral pedal edema, tenderness to palpation on his lower extremities, non-healing ulcers, and a few thick-walled blisters 0.5-2 cm in diameter on the lower extremities, some of which were umbilicated and others that had ruptured, scabbed over, and crusted (Figures 1, 2). Foot X-rays and lower extremity ultrasound were unremarkable, and blood and wound cultures were negative. HIV laboratory results showed improvement, with viral load down to 615 copies/mL and CD4^+^ T-cell count up to 341 cells/µL, suggesting good adherence to antiretroviral therapy. Further diagnostic evaluation was limited by significant procedural anxiety, which resulted in repeated removal of intravenous access, inability to complete MRI imaging of the foot, and deferral of skin biopsy despite ongoing dermatology consultation. His medications were switched to levofloxacin and clindamycin orally to provide broader antimicrobial coverage in the setting of persistent symptoms, and famotidine was added for gastrointestinal protection due to the amount of medication he was currently prescribed.

**Figure 3:**
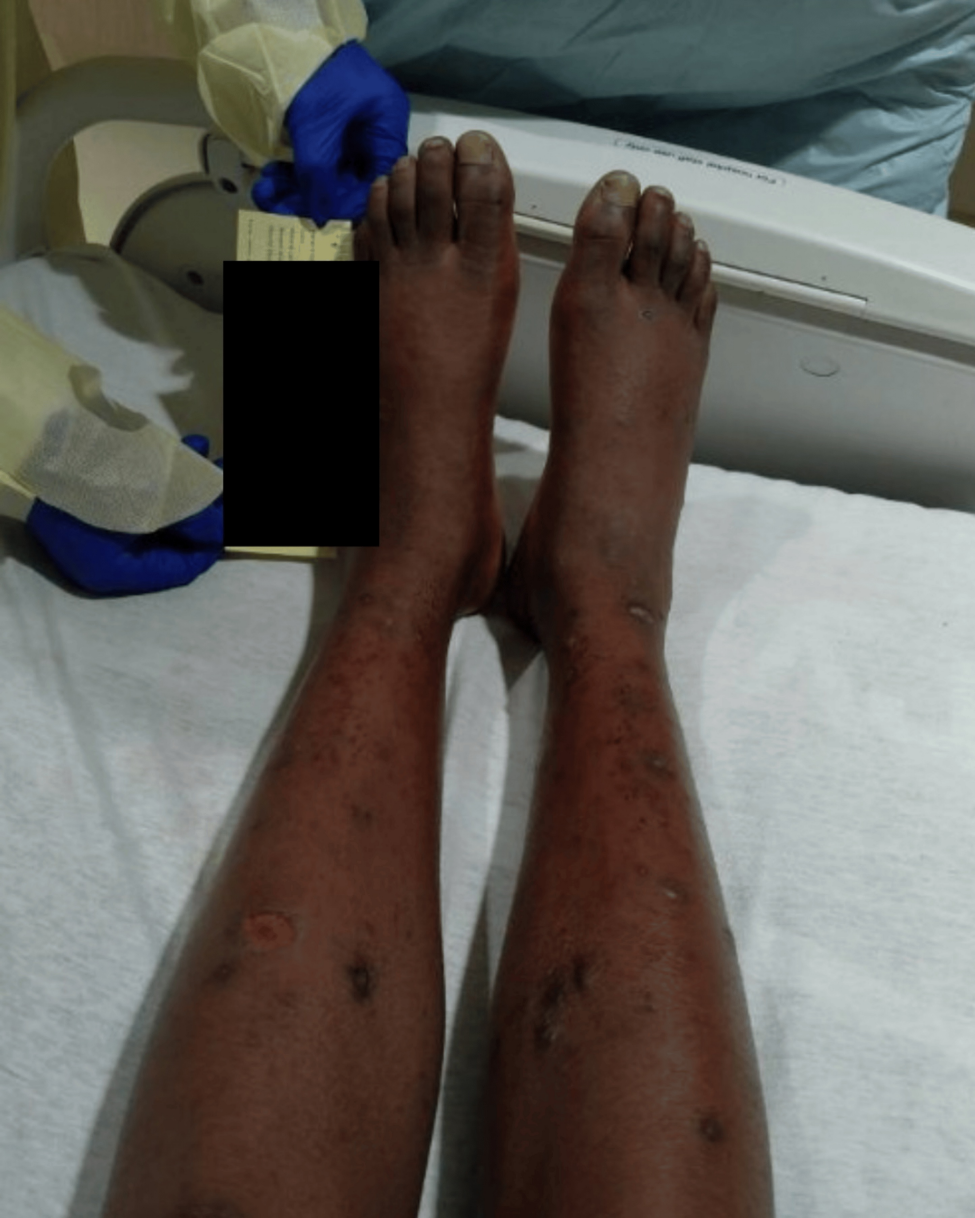
Patient’s petechiae and erythema visible on bilateral lower extremities that started on feet and spread up the ankles. Multiple lesions still present, some crusted over, in various healing stages.

Over the course of his hospitalization, his foot swelling worsened, and dark red petechiae developed on the lower legs and feet (Figure 3). He became unable to ambulate due to the pain and was extremely sensitive to light touch. Despite discussions with the medical team and family, the patient remained anxious and declined further imaging or diagnostic evaluation. Eventually, due to concern for an inconclusive dermatologic progressive infection or systemic immune-mediated process with lack of clinical improvement, the patient's parents and grandmother consented to a skin biopsy against the patient’s wishes. Institutional protocols were followed, with multidisciplinary consultation and discussion, and dermatology obtained a biopsy specimen.

At the time of biopsy, the differential included autoimmune blistering disorders, such as bullous pemphigoid (which may present atypically in immunocompromised hosts), linear IgA dermatosis (commonly drug-induced in children, with Bactrim being a known trigger), reactive arthritis, and erythema multiforme. The patient had also recently developed new petechial and targetoid lesions without mucosal involvement, further complicating the clinical picture.

Following the period around when the biopsy was obtained, the patient’s symptoms began to improve. He reported decreased pain and resumed ambulation. Bactrim was discontinued while awaiting biopsy results due to concern for possible drug-induced hypersensitivity, and a trial of inhaled pentamidine for PJP prophylaxis was attempted but discontinued due to bronchospasm.

Histopathologic analysis of the skin biopsy demonstrated a perivascular lymphocytic infiltrate with eosinophils consistent with a dermal hypersensitivity reaction. Final diagnosis was confirmed when direct cutaneous immunofluorescence demonstrated granular IgA deposition in superficial dermal vessels, with no IgG or IgG4 detected, consistent with IgA vasculitis (Henoch-Schönlein purpura {HSP}).

## Discussion

HIV can have wide-ranging systemic effects and may involve nearly every organ system. While antiretroviral therapy has reduced the incidence of opportunistic infections and acquired immunodeficiency syndrome (AIDS)-defining conditions, both pediatric and adult patients may still experience multisystem manifestations [5]. Constitutional symptoms of acute HIV infection may include fever, fatigue, weight loss, and myalgias, and in addition, dermatologic findings commonly occur in acute disease, which include non-pruritic, erythematous, mononucleosis-like rash typically on the face and trunk. Less frequently, there may be desquamation of the palms and soles, vesiculopustular rashes, or erythema multiforme [5]. These findings are often accompanied by mucosal involvement, which was notably absent in our patient.

HIV is also associated with specific dermatologic conditions. Seborrheic dermatitis may affect up to 80% of HIV-positive individuals, and xerosis, extreme pruritic dryness of the skin, is another common manifestation. Other conditions include bacterial and fungal infections, inflammatory eruptions, and neoplastic lesions, such as Kaposi sarcoma, which presents as violaceous papules or plaques on the skin or mucous membranes [5]. Drug-induced eruptions, particularly secondary to trimethoprim-sulfamethoxazole (TMP-SMX) or name-brand Bactrim, aminopenicillins, and antifungals, are also frequently encountered in HIV-positive patients and typically present as widespread erythematous maculopapular rashes on the trunk and extremities [5].

Some studies have suggested an increased association between HIV and Reiter's syndrome (reactive arthritis), which is often triggered by genitourinary or gastrointestinal infections. This connection is especially relevant in HLA-B27-positive individuals. Although data on this association are mixed, it remained high on the differential in our patient, who was both HIV-positive and HLA-B27 positive and had a concurrent *Yersinia enterocolitica* infection on his first admission, but was then found to be uninfected on his second admission [5,6].

HIV-associated vasculitis is relatively uncommon compared to other systemic manifestations. When it does occur, it is most often in the form of hypersensitivity vasculitis, leukocytoclastic vasculitis, or medium-vessel disease resembling polyarteritis nodosa [7,8]. These conditions can present with purpura, petechiae, peripheral neuropathy, ulceration, or necrosis. Bullous lesions, however, are rarely described [7,8]. In adult patients presenting with purpura or petechiae with or without systemic symptoms, appropriate screening includes hepatitis B and C, HIV testing, and a full rheumatologic workup for inflammatory and autoimmune markers. Such screening is less frequently performed in adolescent patients due to the lower baseline prevalence of these conditions in this age group, which can lead to missing a contributing underlying disease [8].

Although HIV can be associated with several vasculitides, only a few documented cases of IgA vasculitis (Henoch-Schönlein purpura {HSP}) in HIV-positive patients exist, and have been limited to adults [9,10]. One proposed mechanism is that HIV-associated immune dysregulation, including chronic immune activation, may predispose susceptible individuals to IgA-mediated small-vessel vasculitis, although a direct causal relationship has not been established. IgA vasculitis is a small-vessel vasculitis that predominantly affects children, with more than 90% of cases occurring in the pediatric population. It classically presents with palpable purpura, gastrointestinal symptoms, and renal involvement [11]. Compared with previously reported cases of HIV-associated IgA vasculitis, which have been limited to adults with advanced HIV or AIDS, our case has some important distinctions.

Adult cases, such as the 27-year-old man described by Kaya et al., typically present with diffuse palpable purpura involving the trunk, extremities, and palmoplantar surfaces with accompanying high fever, migratory arthritis, lymphadenopathy, cytopenias, and multiorgan involvement [9]. Similarly, another reported adult case involving a 33-year-old man with advanced HIV initially presented with a lower extremity-predominant, non-blanching petechial rash and ankle-localized edema, very similar to our patient’s presentation; however, in that case, symptoms subsequently progressed to widespread purpura with systemic manifestations and biopsy-proven renal involvement [10]. In contrast, our patient presented in early adolescence with a localized lower extremity-predominant rash characterized by bullae, ulcerations, and pedal edema. Our patient remained afebrile and lacked systemic organ involvement. This atypical distribution and morphology contributed to a broad differential focused on blistering disorders, infectious etiologies, and drug-induced processes, delaying diagnosis. Additionally, in our case, psychiatric and behavioral comorbidities significantly delayed diagnostic evaluation, a factor not discussed in other reports. These differences highlight a broader clinical spectrum of HIV-associated IgA vasculitis and show that pediatric patients may present atypically, which can contribute to delayed recognition when HIV is not considered as a diagnosis.

Further complicating dermatologic etiology, our patient had recently started TMP-SMX after his HIV diagnosis, a medication known to trigger hypersensitivity reactions and linear IgA bullous dermatosis [5]. However, his initial dermatologic symptoms preceded initiation of this medication, suggesting that TMP-SMX may have exacerbated his presentation, but did not necessarily initiate the disease process. Ultimately, a skin biopsy demonstrated a perivascular lymphocytic infiltrate consistent with a hypersensitivity reaction, representing the histopathologic correlate of leukocytoclastic vasculitis, followed by direct cutaneous immunofluorescence revealing granular IgA deposition in superficial dermal vessels, leading to a diagnosis of IgA vasculitis. Whether the patient’s HIV-related immune dysregulation played a direct role in the pathogenesis of the vasculitis remains uncertain. This case involved an unusual convergence of an uncommon HIV diagnosis in early adolescence, an atypical presentation of IgA vasculitis, and the possible influence of a commonly prescribed prophylactic drug, creating a diagnostic puzzle without a clearly established causal link.

This case was further complicated by significant psychiatric and behavioral comorbidities, which interfered with timely diagnostics and treatment. The patient refused imaging, laboratory testing, and dermatologic biopsy on multiple occasions and demonstrated inconsistent adherence to medications. These barriers delayed diagnosis and assessment of disease severity. Ultimately, skin biopsy was obtained only after multidisciplinary coordination and caregiver override, and the final diagnosis was reached after multiple visits across a month-long timeframe.

## Conclusions

This case calls attention to the importance of maintaining a broad and evolving differential diagnosis in pediatric and adolescent patients with atypical or multisystem presentations. Although newly acquired HIV is uncommon in this age group and not typically associated with IgA vasculitis, its identification in this patient reframed the diagnostic approach and prompted urgent management given his CD4^+^ T-cell count nearing the AIDS-defining threshold. Whether HIV contributed directly to the vasculitic process or occurred coincidentally remains uncertain; however, its discovery proved clinically significant and influenced subsequent evaluation. This case highlights how diagnostic delay may be compounded by atypical disease morphology, low perceived disease prevalence, and medical or social complexity. Clinicians should avoid narrowing the differential diagnosis based solely on age, presumed risk, or statistical prevalence and should consider an early workup for infectious, autoimmune, or inflammatory causes when initial evaluations are unrevealing. Maintaining diagnostic flexibility and using a full range of diagnostic tools, even when presentations deviate from classic patterns, may uncover important but unexpected conditions.
